# Case Report: A case of persistent fifth aortic arch associated with stenosis and interrupted aortic arch

**DOI:** 10.3389/fped.2025.1676467

**Published:** 2025-11-27

**Authors:** Gao Yi, Hu Zehang, Fan Shumin

**Affiliations:** Department of Ultrasound, Shenzhen Children’s Hospital, Shenzhen, China

**Keywords:** congenital heart disease, persistent fifth arch, aortic arch interruption, echocardiography, children

## Abstract

Persistent fifth aortic arch (PFAA) is a rare congenital cardiovascular anomaly, often associated with other defects such as interrupted aortic arch (IAA) or patent ductus arteriosus (PDA). We report a 12-day-old male neonate presenting with respiratory distress, edema, and oliguria. Physical examination revealed a grade 2/6 systolic murmur at the left sternal border. Echocardiography identified PFAA with coarctation, IAA, PDA, and an atrial septal defect (ASD), confirmed by cardiac CT. Surgical intervention included aortic arch repair, fifth arch excision, and ASD closure. The postoperative recovery was smooth, and the patient remained clinically well with normal cardiac function during the 1-year follow-up period. This case underscores the critical role of echocardiography in early diagnosis and surgical planning for complex congenital heart disease.

## Introduction

Persistent fifth aortic arch (PFAA) arises from incomplete embryonic regression of the fifth aortic arch, often coexisting with cardiovascular anomalies such as PDA, IAA, or pulmonary atresia (1, 2). Gupta et al. (3) defined PFAA as an extrapericardial vessel bridging the ascending and descending aorta. Histologically distinct from normal aortic tissue (4), PFAA results from failed degeneration of the fifth branchial arch, contributing to vascular ring formation and hypoplasia (5–7). Clinical manifestations depend on associated defects and hemodynamic impact ^(^8). While asymptomatic cases may occur incidentally (2), PFAA with coarctation often presents with hypertension, pulse discrepancies, or heart failure (9). This report details a rare case of type B PFAA with IAA, emphasizing the utility of multimodal imaging in diagnosis and management.

## Case report

A 12-day-old male was admitted for progressive respiratory distress, edema, and oliguria (urine output: 0.3 mL/kg/h). Born vaginally at 39 + 3 weeks, he had been hospitalized previously for persistent irritability and received respiratory suctioning, monitoring, and antibiotics (amoxicillin) before discharge. Three days later, he developed recurrent respiratory distress, necessitating Nasal Continuous Positive Airway Pressure (NCPAP) and readmission. On admission, physical examination revealed pitting edema over the lower back, a grade 2/6 systolic murmur at the left sternal border (2nd–3rd intercostal spaces), and hepatomegaly (3 cm below the costal margin). Upper limb blood pressure was 85/40 mmHg (lower limbs unmeasured). Oxygen saturation (SpO_2_) was 99% in all limbs. Laboratory findings included hypoalbuminemia (26.9 g/L; normal range: 35–50 g/L) and elevated N-terminal pro-BNP (>25,000 pg/mL; normal: <300 pg/mL).

**Figure 1:**
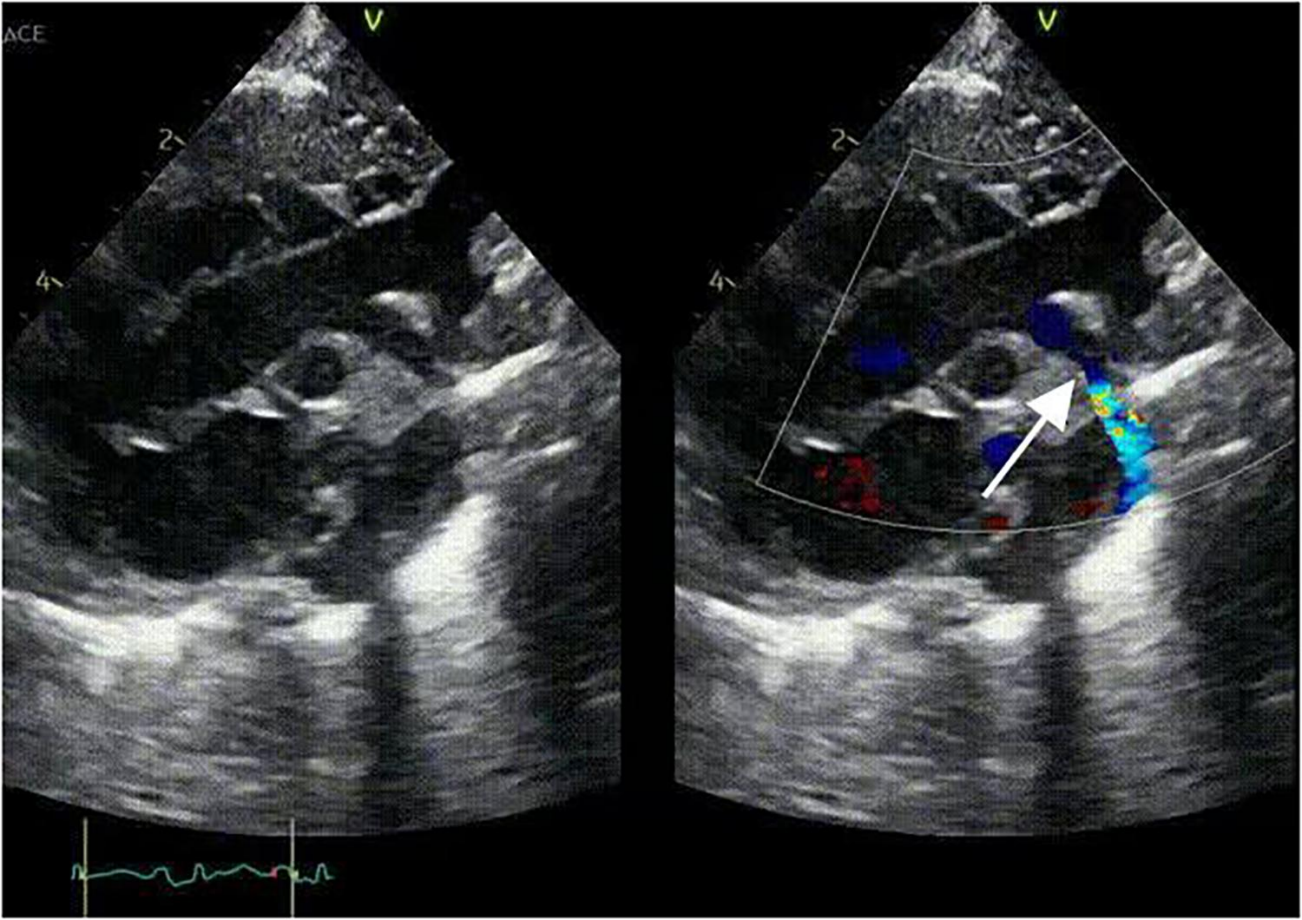
2D-TTE: persistent fifth aortic arch associated with stenosis (indicated by arrows) and an interrupted fourth aortic arch.

**Figure 2:**
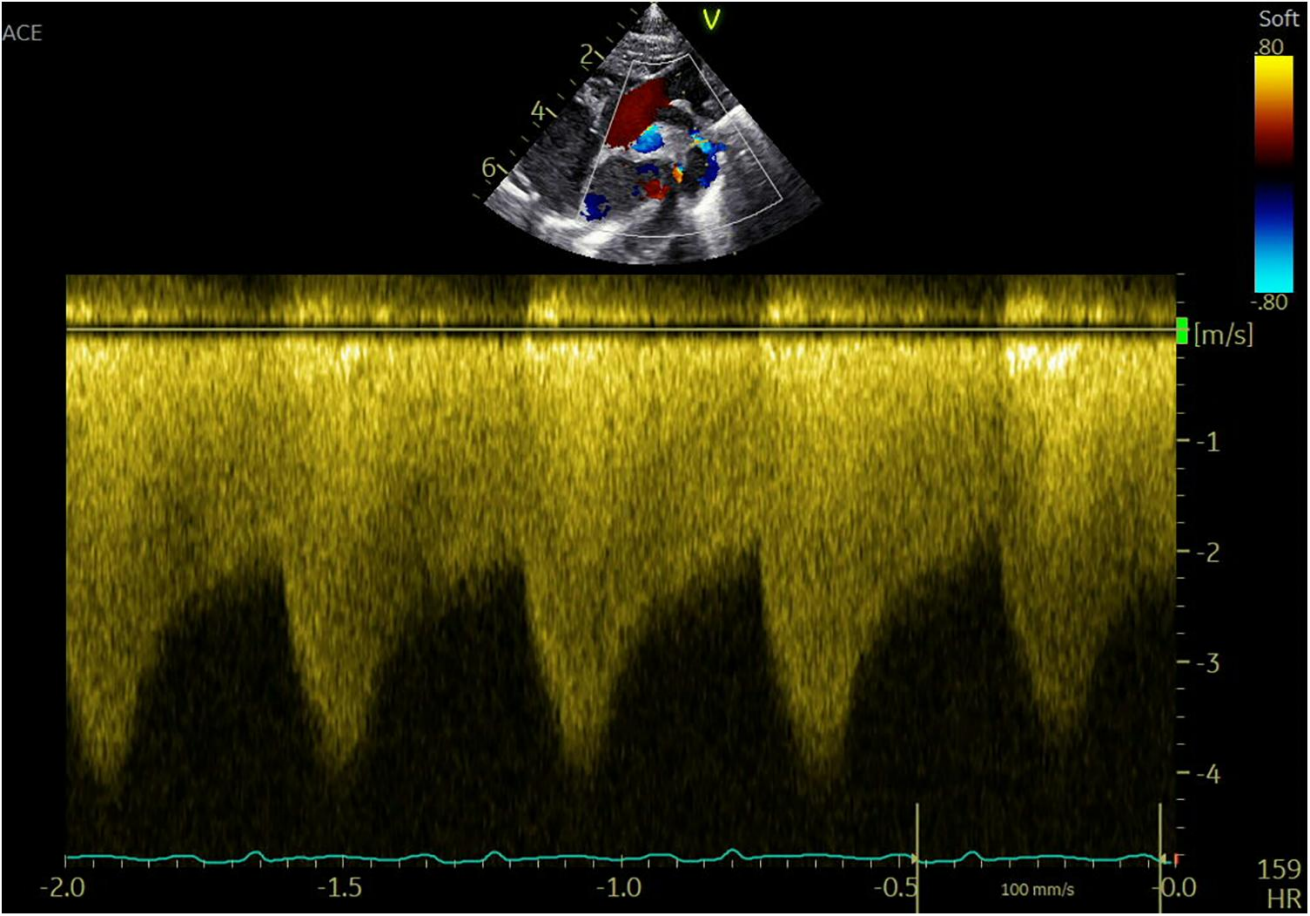
Color Doppler demonstrating turbulent flow at the coarctation site (peak velocity: 4.1 m/s).

**Figure 3:**
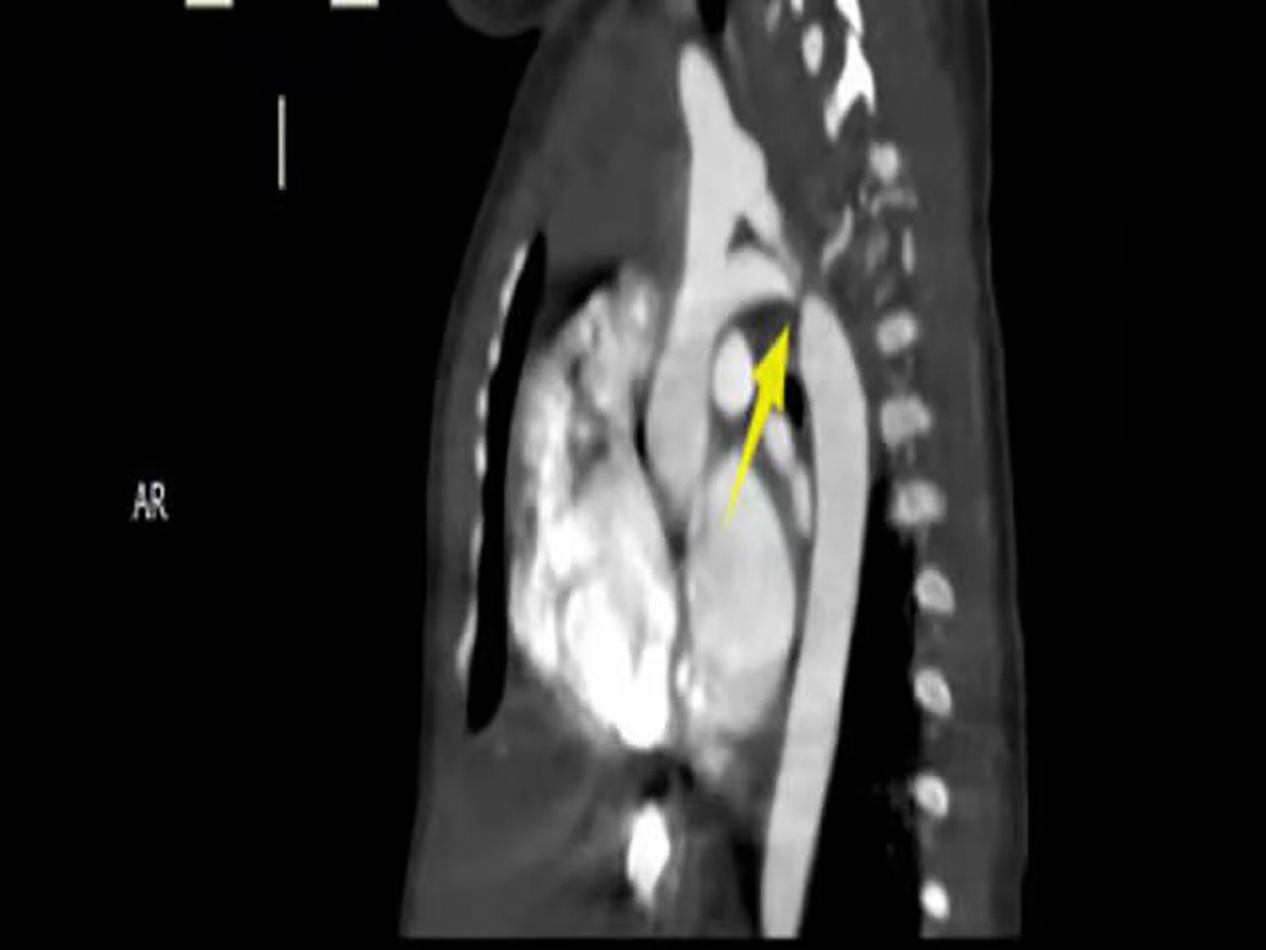
CTA: persistent fifth aortic arch associated with stenosis (indicated by arrows) and an interrupted fourth aortic arch. The fourth arch was interrupted at the distal end of the left subclavian artery, about 12 mm long. The fifth arch continued with the descending aorta, with a length of about 9 mm and an internal diameter of about 5 m at the proximal end.

**Figure 4:**
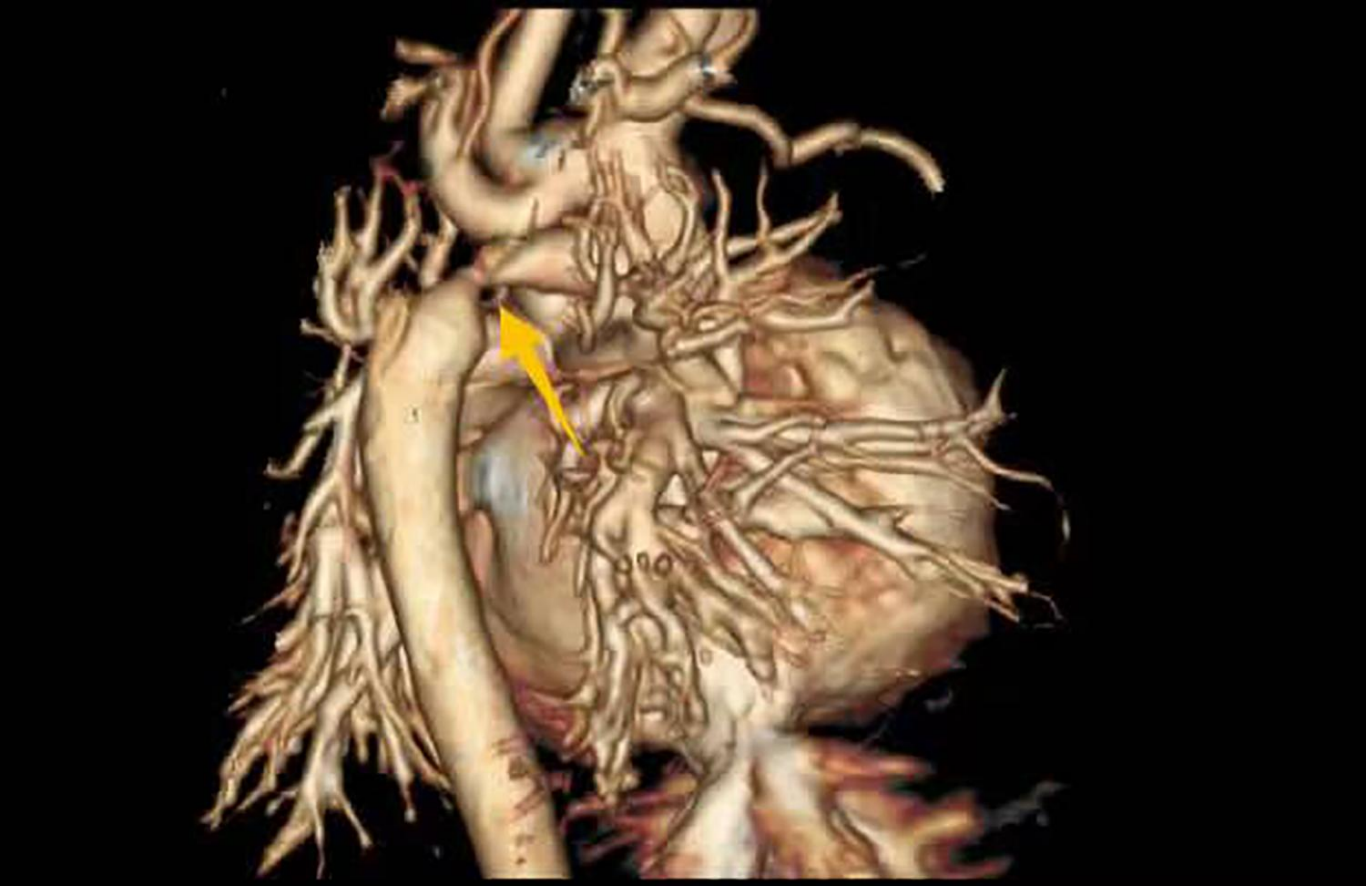
CT 3D reconstruction: persistent fifth aortic arch associated with stenosis (indicated by arrows) and an interrupted fourth aortic arch.

Imaging studies included a chest x-ray showing cardiomegaly and pulmonary congestion, followed by echocardiography, which identified a persistent fifth aortic arch (PFAA) with coarctation (narrowest diameter: 5 mm), an interrupted fourth aortic arch (Figure 1). Color Doppler demonstrating turbulent flow at the coarctation site (peak velocity: 4.1 m/s) (Figure 2). A patent ductus arteriosus (PDA; 3 mm) located between the proximal descending aorta and the pulmonary artery bifurcation, and an atrial septal defect (ASD; 6 × 8 mm), accompanied by reduced left ventricular systolic function (ejection fraction: 49%). Cardiac CT (Figures 3, 4) further confirmed the anatomical details, including a 12 mm gap in the fourth aortic arch and the PFAA's aberrant course.

Initial stabilization involved nasal continuous positive airway pressure (NCPAP), intravenous dopamine to address cardiogenic shock, albumin supplementation, and diuretics. Definitive surgical management included aortic arch repair under cardiopulmonary bypass, involving excision of the fifth arch, end-to-end anastomosis between the native arch and descending aorta, PDA ligation, and ASD closure. Postoperative imaging confirmed restored aortic flow and normalized cardiac function (LVEF: 69%), with the patient discharged symptom-free on postoperative day 11.

## Discussion

Persistent fifth aortic arch (PFAA) represents an exceptionally rare congenital cardiovascular anomaly with complex embryological origins. During mammalian cardiovascular development, six pairs of aortic arches sequentially form between embryonic weeks 3–8, undergoing precise regression and remodeling processes to establish definitive vascular structures (10). PFAA arises from the failure of fifth branchial arch regression, resulting in persistent vascular channels that exhibit distinct histological characteristics compared to normal aortic tissue (4). This anomaly may manifest as three anatomical subtypes: systemic-to-systemic connections (types A and B) or systemic-to-pulmonary shunts (type C) (11). In type B PFAA, as observed in this case, the fourth aortic arch is interrupted or atretic, leaving the fifth arch as the sole conduit between the ascending and descending aorta (12). The hemodynamic consequences of this configuration depend critically on associated anomalies, such as coarctation or ductal patency, which collectively determine clinical presentation and urgency of intervention.

The neonate in this report exhibited classic manifestations of type B PFAA complicated by coarctation and interrupted fourth aortic arch: respiratory distress, systemic edema, and oliguria. These symptoms reflect the pathophysiological cascade of left ventricular pressure overload, impaired systemic perfusion, and subsequent cardiogenic shock. Notably, the absence of lower limb blood pressure measurement at initial evaluation delayed recognition of coarctation—an oversight that underscores the necessity for systematic four-limb pressure assessment in neonates with suspected aortic arch anomalies. This case aligns with previous reports (8, 9) emphasizing that PFAA-associated coarctation often presents with subtle but critical perfusion discrepancies, requiring heightened clinical suspicion.

Diagnostically, this case illustrates the complementary roles of echocardiography and advanced imaging. While echocardiography provided initial identification of PFAA morphology, coarctation severity, and associated defects (PDA, ASD), cardiac CT offered three-dimensional anatomical clarification of the 12 mm fourth arch interruption and fifth arch course. Current guidelines (13, 14) endorse this multimodal approach, as echocardiography alone may inadequately visualize cervical vasculature or complex arch relationships. Although digital subtraction angiography (DSA) and cardiac catheterization remain gold standards for anatomical delineation (15), their invasive nature and radiation exposure limit utility in critically ill neonates. The noninvasive paradigm demonstrated here—combining echocardiography for functional assessment and CT for anatomical precision—provides a safer diagnostic framework without compromising accuracy.

Surgical management of PFAA with hemodynamically significant anomalies demands meticulous anatomical reconstruction. In this patient, the surgical strategy addressed three critical components: (1) excision of the hypoplastic fifth arch to eliminate turbulent flow, (2) end-to-end anastomosis restoring aortic continuity, and (3) concurrent closure of PDA and ASD to normalize circulatory physiology. This approach aligns with Zhao et al.'s principles (10) for PFAA repair, prioritizing anatomical correction while minimizing residual gradients. The postoperative ejection fraction improvement (49%–69%) and symptom resolution validate the intervention's success, though long-term surveillance remains essential to monitor for recoarctation or ventricular remodeling.

Two broader implications emerge from this case. First, PFAA's rarity and phenotypic variability necessitate high-volume center management, where multidisciplinary teams can integrate advanced imaging, neonatal critical care, and specialized surgical expertise. Second, the evolving role of fetal echocardiography warrants emphasis—earlier prenatal detection could enable delivery planning and immediate postnatal stabilization, potentially mitigating complications like renal hypoperfusion observed here. Future studies should explore optimal timing for surgical intervention in asymptomatic PFAA cases, balancing the risks of early intervention against the consequences of delayed treatment.

In conclusion, this case reinforces PFAA as a diagnostically and therapeutically challenging entity within congenital heart disease. It highlights the critical interplay between meticulous clinical examination, strategic imaging utilization, and tailored surgical correction. As neonatal care advances, continued refinement of diagnostic protocols and surgical techniques will be paramount to improving outcomes for this rare patient population.

## Data Availability

The original contributions presented in the study are included in the article/Supplementary Material, further inquiries can be directed to the corresponding author.
